# Analgesic safety and efficacy of regional anesthetic blocks in anterior lumbar interbody fusion surgery: A systematic review with meta-analyses

**DOI:** 10.1097/MD.0000000000043787

**Published:** 2025-08-29

**Authors:** Luke J. Weisbrod, Anthony J. Maxin, Judith R. Bergjord, Andrew P. Gard, Daniel L. Surdell

**Affiliations:** a Department of Neurosurgery, University of Nebraska, Omaha, NE; b School of Medicine, Creighton University, Omaha, NE; c Health Sciences Library, Creighton University, Omaha, NE; d Department of Neurosurgery, MD West One, Omaha, NE.

**Keywords:** anterior, block, lumbar, rectus abdominis, transversus abdominis

## Abstract

**Background:**

Anterior regional anesthetic blocks have been utilized as a part of a multi-modal pain regimen in patients undergoing abdominal surgery. Recently, studies have investigated their safety and efficacy in anterior lumbar interbody fusion (ALIF) surgery.

**Methods:**

A systematic literature search was conducted through MEDLINE, Embase, and Cochrane Library on January 30, 2024, for articles investigating the safety and efficacy of preoperative regional blocks in patients undergoing ALIF surgery compared to patients who did not receive a preoperative regional block. Meta-analyses were performed to calculate the pooled mean difference with 95% confidence intervals (CI) using a random-effects model. Criteria for inclusion were patients undergoing ALIF surgery; sample size of ≥ 5 patients in each study group; patient population that pre-operatively received an anterior abdominal regional block; patient population age ≥ 18 years; available data regarding postoperative numerical pain scores and opioid requirements. Studies with overlapping patient data already included for the analysis and lack of pre-specified data were excluded.

**Results:**

Four studies were included in the pooled analysis with a total number of 415 patients. The results of the pooled analysis showed that in patients undergoing ALIF surgery, a preoperative regional block resulted in a decrease in opioid requirements at 48 hours post-operatively (MD −0.37, 95% CI [−0.63 to −0.11], *I*^2^ = 6.38%, *P *= .005) and 72 hours post-operatively (MD −0.76, 95% CI [−1.22 to −0.29], *I*^2^ = 78.48%, *P* = .002). A preoperative regional block resulted in a decrease in numerical pain rating scores at 24 hours post-operatively (MD −0.52, 95% CI [−1.16 to 0.13], *I*^2^ = 84.97%, *P *= .116) and a decrease in opioid requirements at 24 hours post-operatively (MD −0.44, 95% CI [−1.60 to 0.72], *I*^2^ = 94.37%, *P* = .462), though not to a level of statistical significance.

**Conclusion:**

This review suggests that regional blocks in ALIF surgery are reasonably safe and result in improved analgesic efficacy. This study is limited by the pooled data from relatively small series, many of which were retrospective in design. Robust prospective, randomized studies are necessary to help inform the safety and efficacy of regional blocks for ALIF surgery.

## 1. Introduction

Anterior lumbar interbody fusion (ALIF) is a surgical procedure that has evolved to be an efficacious surgical technique, particularly within the realm of discogenic back pain, as well as the revision of failed posterior fusion.^[1]^ The anterior retroperitoneal approach facilitates access to the ventral surface of the exposed disc, allowing for efficient discectomy and direct implant insertion. The anterior access permits maximization of the implant size and surface area, facilitating correction of lordosis and indirect foraminal decompression secondary to foraminal height restoration. The ALIF approach is most suitable for the L5-S1 level caudal to the aorta and inferior vena cava bifurcations, and less so at L4-L5 and more rostral levels due to the obstructing vascular anatomy.^[2]^ Less invasive procedures, such as the ALIF approach, allow for sparing of posterior spinal musculature dissection. The ALIF approach remains a moderately painful surgery associated with significant postoperative opioid requirements that may prolong hospital length of stay and hinder recovery after surgery.^[3]^

Postoperative pain management presents a significant challenge, particularly in the elderly population who are more sensitive to the adverse effects of opioid analgesia.^[4]^ In 2020 the elderly population ≥ 65 years of age comprised 16.8% of the United States of America (USA) population, a number that is expected to continue growing.^[5]^ Opioid analgesia can result in multiple adverse effects including respiratory depression, urinary retention, constipation, and nausea/vomiting, all of which become magnified in the aging population.^[6]^ Furthermore, opioids can become addictive as has been observed in the opioid epidemic with an estimated 3% of opioid naïve patients continuing to use opioids for more than 90 days following major elective surgery.^[7]^

Given the anterior approach of ALIF surgery, these patients could benefit from anterior regional anesthetic blocks, such as transversus abdominis plane (TAP) or rectus sheath blocks for postoperative pain management to decrease postoperative opioid use. TAP blocks are plane-based regional anesthetic blocks under ultrasound guidance^[8]^ that have been utilized as part of a multi-modal approach to pain control in patients undergoing abdominal surgeries such as appendectomy, abdominoplasty, hysterectomy, and cesarean section.^[9]^ Like the TAP block, the rectus sheath block is a safe preoperative ultrasound-guided procedure for providing anterior abdominal wall analgesia, typically with more efficacy near the midline.^[10–12]^

Recently, studies have investigated the feasibility of anterior regional anesthetic blocks for ALIF surgery.^[13,14]^ The purpose of this systematic review with meta-analyses is to determine the safety and analgesic efficacy of preoperative regional anesthetic blocks in patients undergoing ALIF surgery.

## 2. Materials and methods

### 2.1. Search strategy

A comprehensive, systematic literature search was conducted through MEDLINE, Embase, and Cochrane Library on January 30, 2024, for articles investigating the safety and efficacy of preoperative regional anesthetic blocks compared to control for patients undergoing ALIF surgery with adherence to Preferred Reporting Items for Systematic Reviews and Meta-Analyses (PRISMA) guidelines for reporting of the results.^[15]^ The search strategies included titles and keywords that included the search concepts: ALIF or ALIF, rectus sheath block, and TAP or TAP block (Table 1). We focused the search on work with adult patients by first removing articles indexed as concerning nonhuman animals if these were not also indexed as concerning humans and then removing articles indexed as concerning pediatric age groups if these were not also indexed as concerning adults. Conference abstracts, book chapters, and clinical registry records were excluded. This study was registered on Prospero and assigned ID number CRD42024521089. Ethical approval from our local institutional review board was not necessary as our study was a review of the existing literature.

**Table 1:** Search strategies.

#	Query	Limiters/expanders	Last run via	Results
S9	S5 AND S8	*Expanders – apply equivalent subjects Search modes – Boolean/phrase	**Interface – EBSCOhost Research databases Search screen – advanced search Database—MEDLINE complete	65
S8	S3 OR S7	*	**	27,234
S7	Nerve Block	*	**	26,362
S6	S3 AND S5	*	**	8
S5	S4 OR Anterior, Lumbar, Fusion OR (ALIF) OR LLIF	*	**	32,995
S4	Spinal Fusion	*	**	32,271
S3	S1 OR S2	*	**	1817
S2	Rectus, Sheath, Block	*	**	265
S1	Transverse, Abdomin, Block OR TAP Block OR Transforaminal Abdomin	*	**	1623
Cochrane	Transverse, Abdomin, Plane Block OR Transforaminal Abdominal Plane Block OR TAP Block AND Anterior Lumbar Interbody Fusion OR ALIF OR LLIF	*	Cochrane Libraries	4
E1	Transversus Abdominis Plane Block OR TAP Block OR Transforaminal AND Abdominal AND Plane AND Block OR Transverse, Abdomin, Plane Block	*	Embase	3018
E2	Rectus Sheath Block OR Nerve Block	*	Embase	46,012
E3	E1 or E2	*	Embase	48,222
E4	Anterior Lumbar Interbody Fusion OR Lateral Lumbar Interbody Fusion OR ALIF OR LLIF	*	Embase	3059
E5	E3 AND E4	*	Embase	20

ALIF = anterior lumbar interbody fusion, E = embase search strategy, LLIF = lateral lumbar interbody fusion, S = MEDLINE search strategy, TAP = transversus abdominis plane.

### 2.2. Selection criteria

We included all English-language articles that evaluated the safety and efficacy of preoperative regional anesthetic blocks in patients undergoing ALIF surgery in comparison to controls who received general anesthesia only or a placebo followed by a standard postoperative pain regimen. The criteria for inclusion in the study were patients undergoing ALIF surgery; a sample size of ≥ 5 patients in each study group; a patient population that pre-operatively received an anterior abdominal regional block; adult patient population with age ≥ 18 years; available data regarding the duration of surgery, length of hospital stay, postoperative numerical pain scores, postoperative opioid requirements and adverse events. Studies with overlapping patient data already included for the analysis and lack of pre-specified data were excluded.

### 2.3. Outcomes

The outcomes of interest in this study included the duration of surgery, length of hospital stay, postoperative numerical pain scores, postoperative opioid requirements, and adverse events.

### 2.4. Data extraction

Data were extracted independently by 2 researchers (LW and AM) and were collected using Microsoft Excel (Microsoft Corp., Redmond, Washington). We recorded the following information: last name of the first author and year of study, location in which the study occurred, number of patients included in the study, median age, gender ratio, type of regional anesthetic block and dose of administered medications, control group intervention, surgical details including the number of spinal levels fused and if constructs were supplemented with posterior instrumentation, the type of baseline analgesia, postoperative numerical pain rating scores, postoperative opioid requirements, duration of surgery, hospital length of stay and adverse events.

### 2.5. Quality assessment

The Newcastle-Ottawa scale was used to assess the quality of included studies that did not have a prospective, randomized design.^[16]^ Two reviewers, (LW and AM) performed the quality assessments individually, and any discrepancies were resolved with discussion. Studies rated with 0 to 3 stars were considered low-quality, studies with 4 to 6 stars were considered medium-quality, and studies with 7 to 9 stars were considered high-quality.

### 2.6. Statistical analysis

Meta-analyses were performed on outcomes of interest if ≥3 study populations were available for pooled analysis of designated outcomes of interest. Meta-analyses were performed to calculate the pooled mean difference with 95% confidence intervals (CI) and prediction intervals (PI) using a random-effect model given anticipated heterogeneity amongst included studies. Statistical significance was achieved with a *P*-value < .05. Results are presented in forest plots. All analyses were completed using the meta-analysis functions in the open statistical software Python version 3.10 and Jamovi version 2.4.7.

## 3. Results

### 3.1. Search results

**Figure 1.**
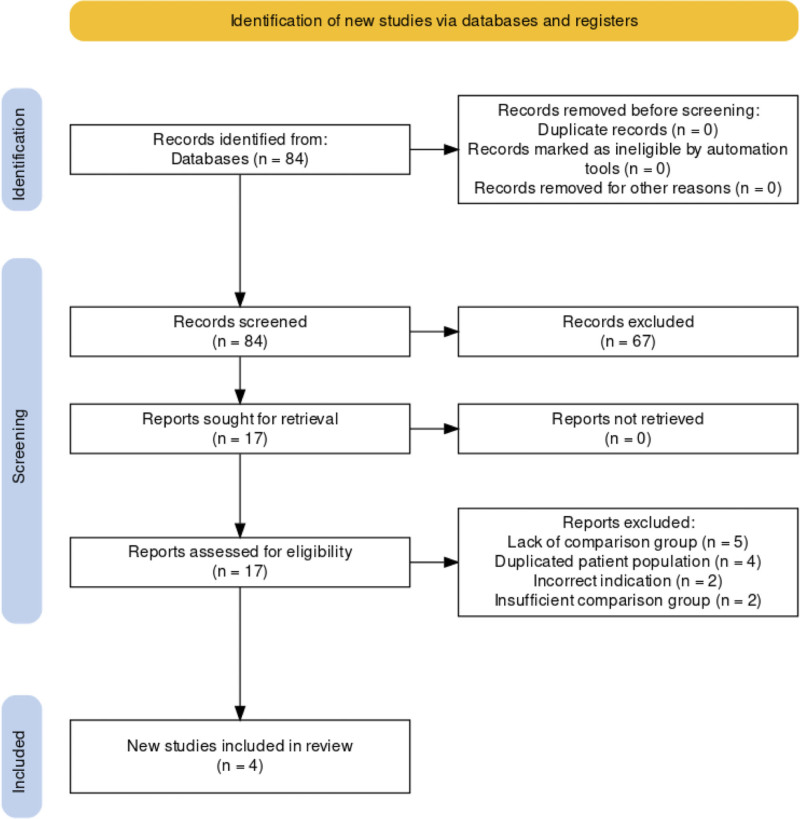
Preferred reporting items for systematic reviews and meta-analyses flowchart.

MEDLINE, Embase, and Cochrane Library databases identified 84 publications (Fig. 1). Two independent researchers (LW and AM) screened the 84 articles identified by the search strategy. Sixty-seven articles were excluded during the initial screening and 17 articles were assessed for eligibility. Of these 17 articles, 4 were included for analysis and 13 were excluded. Reasons for exclusion included: lack of comparison group (5), duplicated patient population (4), incorrect indication (2), and insufficient comparison group (2). In the final meta-analyses, 4 studies were included after studies that failed to meet inclusion criteria were removed. The characteristics of the 4 studies included in the meta-analyses are presented in Table 2.^[17–20]^ Three studies were performed in the USA and 1 study was performed in France. Three studies were retrospective, and 1 study was prospective. The control comparison group was general anesthesia in 3 studies and a placebo in 1 study. The specific type of regional anesthetic block was a TAP block in 3 studies and a TAP in addition to rectus sheath block in 1 study. The medication regimen and dosing were heterogenous amongst the studies and included 0.5% ropivacaine in 2 studies, 0.5% bupivacaine in 1 study, and 0.25% bupivacaine with 1:400,000 epinephrine and 5 milligrams (mg) dexamethasone in 1 study. The quality assessment results as measured by the Newcastle-Ottawa Scale score was 7 in 2 studies, 8 in 1 study and not applicable in 1 study due to its prospective nature (Table 3).

**Table 2:** Characteristics of studies included in meta-analyses.

Study	Study year	Study design	Study location	NOS	Type of block	Medication (s) in block	Control
Esmende^[17]^	2022	R	France	7	TAP, b/l rectus sheath	30 mL bupivacaine 0.25%, 1:400,000 epinephrine, 5 mg dexamethasone	No block; GA only
Colon^[18]^	2023	R	USA	8	TAP	25–30 mL 0.5% bupivacaine	No block; GA only
Coquet^[19]^	2023	PR	USA	NA	b/l TAP	15 mL ropivacaine 0.5% (5 mg/mL)	Placebo
Ogura^[20]^	2020	R	USA	7	b/l TAP	0.5% ropivacaine	No block; GA only

b/l = bilateral, GA = general anesthesia, mg = milligram, mL = milliliter, NA = not applicable, NOS = Newcastle-Ottawa score, PR = prospective randomized, R = retrospective, TAP = transversus abdominis plane, USA = United States of America.

**Table 3:** Results of quality assessment by Newcastle-Ottawa scale.

Study	D1 (1)	D2 (1)	D3 (1)	D4 (1)	D5 (2)	D6 (1)	D7 (1)	D8 (1)	Overall (9)
Esmende^[17]^	0	1	1	1	1	1	1	1	7
Colon^[18]^	1	1	1	1	2	1	1	0	8
Coquet^[19]^	–	–	–	–	–	–	–	–	N/A
Ogura^[20]^	0	1	1	1	1	1	1	1	7

D1) Representativeness of the exposed cohort, D2) selection of the nonexposed cohort, D3) ascertainment of exposure, D4) demonstration that the outcome of interest was not present at start of study, D5) comparability of cohorts on the basis of the design or analysis, D6) assessment of outcome, D7) was follow-up long enough for outcomes to occur, D8) adequacy of follow-up of cohorts.

D = domain; N/A = not applicable.

The meta-analyses included 415 patients whose demographics are included in Table 4. Of the 415 total patients, 221 received a preoperative regional anesthetic block. The control groups included 194 patients. The range of the median age of the preoperative regional anesthetic block and control groups was 46 to 58 and 50 to 64 respectively. The range of male-to-female gender ratios of the preoperative regional anesthetic block and control groups ranged from 0.96 to 1.51 and 0.79 to 2.00 respectively. The number of levels of instrumentation was 1 to 2 in 2 studies and 1 to 3 in 2 studies. In 3 studies, patients were included who also underwent posterior instrumentation. The baseline postoperative analgesic regimens were heterogenous, including oxycodone and hydromorphone in 1 study, acetaminophen, cyclobenzaprine, and gabapentin in 1 study, and paracetamol, nefopam, ketoprofen, and morphine in 1 study. One study did not provide the baseline postoperative analgesic regimen.

**Table 4:** Characteristics of patient demographics included in meta-analyses.

Study	N Block	N Control	Mdn age block	Mdn age control	Gender ratio M:F block	Gender ratio M:F control	Level (s); *P*?	Baseline analgesia
Esmende^[17]^	88	87	53.1	52.5	1.51	1.64	1–3; N	Oxycodone, hydromorphone
Colon^[18]^	47	52	58	64	0.96	1.48	1–2; Y	NA
Coquet^[19]^	18	16	46	50	1.33	2.00	1–3; Y	Paracetamol, nefopam, ketoprofen, morphine PCA × 24 h followed by PO opioid
Ogura^[20]^	68	39	56.8	51.7	1.17	0.79	1–2; Y	Acetaminophen, cyclobenzaprine, gabapentin

Level(s), number of levels fused during ALIF surgery.

F = female, M = male, Mdn = median, N = number, NA = not available, P = posterior instrumentation, PCA = patient-controlled analgesia, PO = per os, Y = yes.

### 3.2. Meta-analyses

There was a sufficient number of study populations to perform meta-analyses for numerical pain rating scores at 24 hours post-operatively, as well as the postoperative opioid requirements at 24 hours, 48 hours, and 72 hours post-operatively.

### 3.3. Numerical pain rating score 24 hours post-operatively

**Figure 2.**
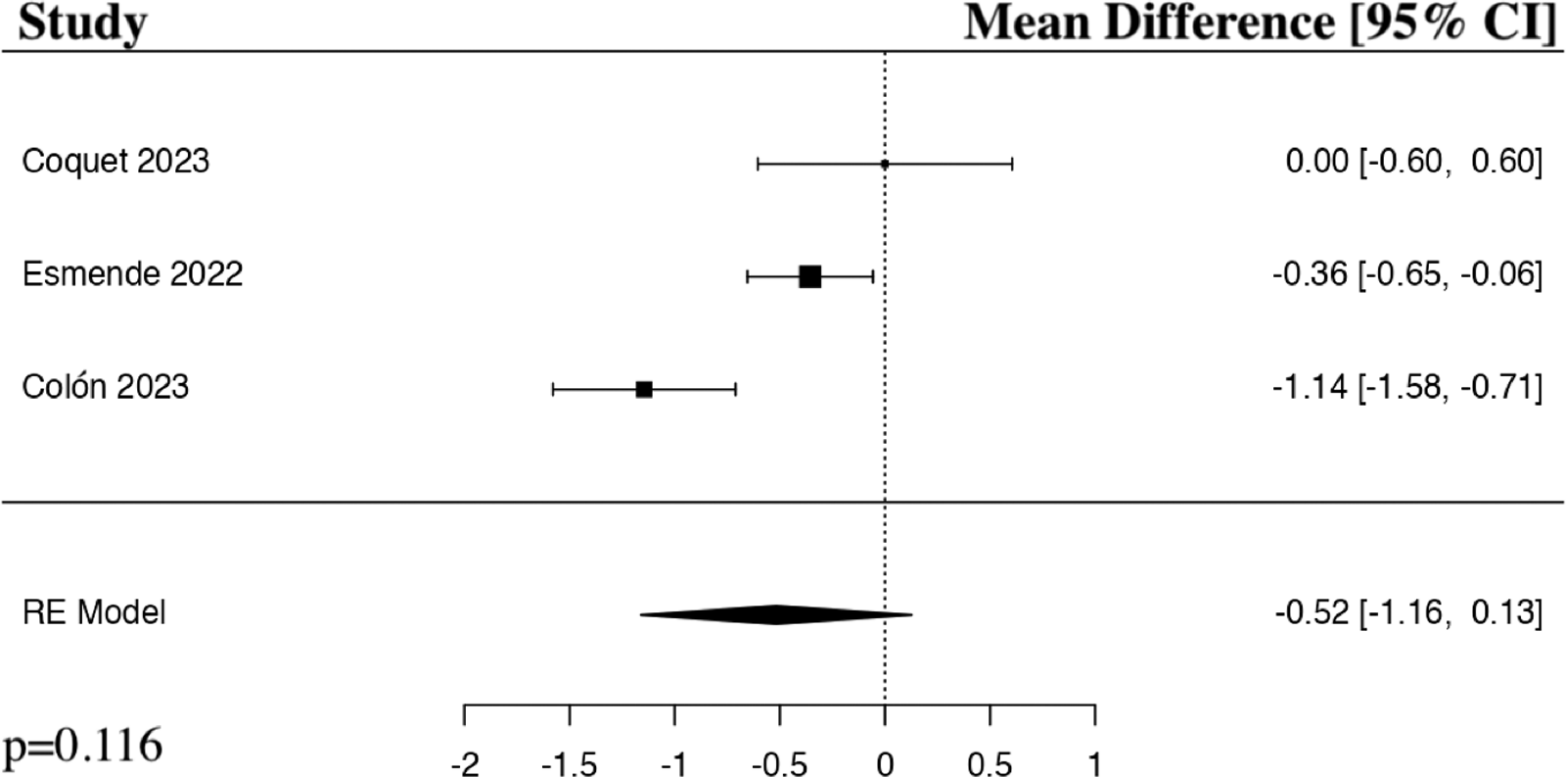
Forest plot demonstrating random-effects model for numerical pain rating scores 24 hours post-operatively in patients who received a regional anesthetic block in comparison to controls for anterior lumbar interbody fusion spine surgery. CI = confidence interval, RE = random effects.

The meta-analysis for the numerical pain rating score at 24 hours post-operatively included 3 studies.^[17–19]^ The results of the pooled analysis showed that a preoperative regional anesthetic block resulted in a decrease in numerical pain rating scores at 24 hours post-operatively in comparison to controls in patients undergoing ALIF surgery, though not to a level of statistical significance (MD −0.52, 95% CI [−1.16 to 0.13], PI [−1.50 to 0.39], *I*^2^ = 84.97%, *I*^*2*^ 95% CI [0.00%–87.47%], *P *= .116) (Fig. 2).

### 3.4. Opioid requirements 24 hours post-operatively

**Figure 3.**
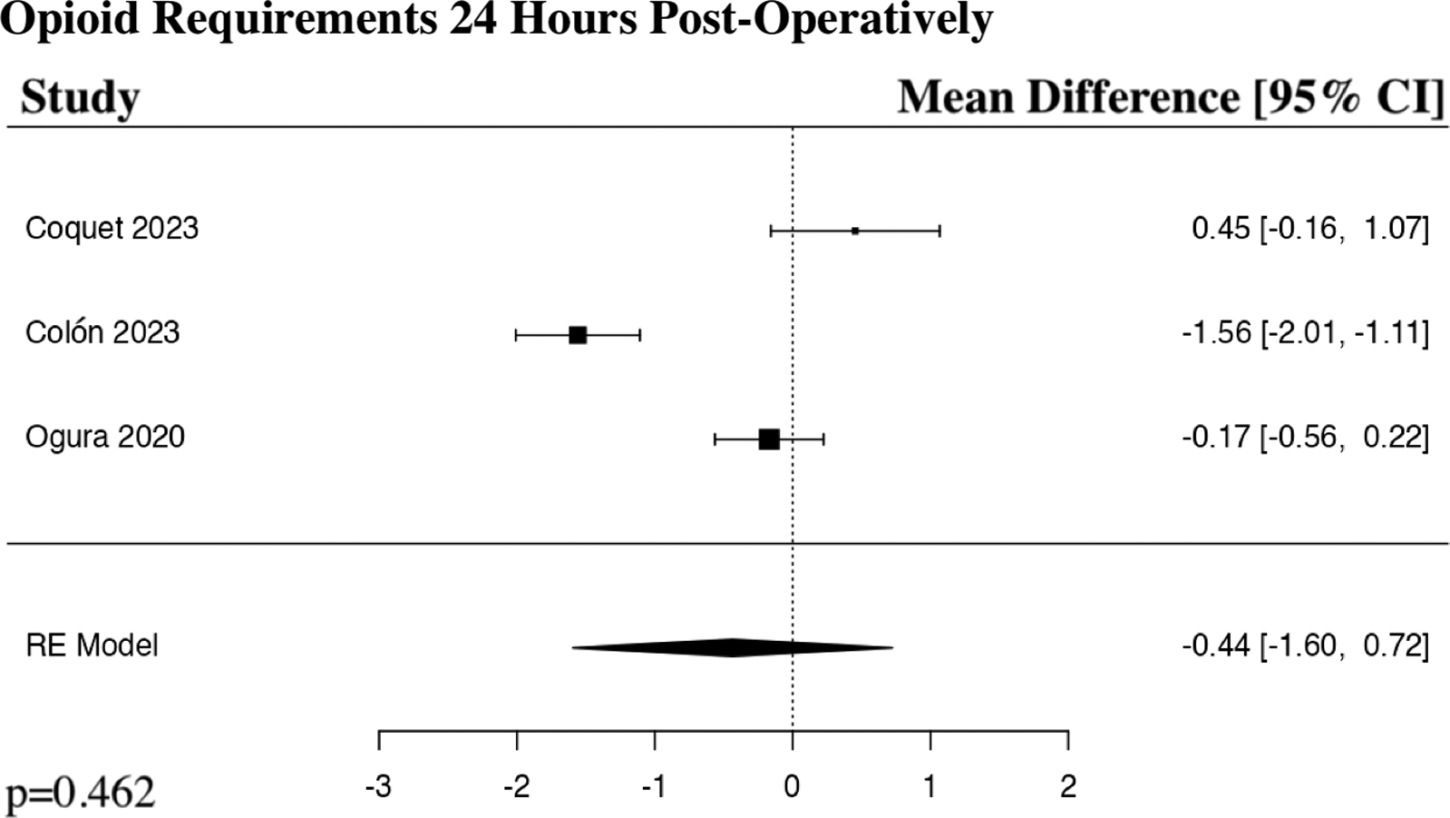
Forest plot demonstrating random-effects model for opioid requirements 24 hours post-operatively in patients who received a regional anesthetic block in comparison to controls for anterior lumbar interbody fusion spine surgery. CI = confidence interval, RE = random effects.

The meta-analysis for the opioid requirements at 24 hours post-operatively included 3 studies.^[18–20]^ The results of the pooled analysis showed that a preoperative regional anesthetic block resulted in a decrease in opioid requirements at 24 hours post-operatively in comparison to controls in patients undergoing ALIF surgery, though not to a level of statistical significance (MD −0.44, 95% CI [−1.60 to 0.72], PI [−2.65 to 1.77], *I*^2^ = 94.37%, *I*^*2*^ 95% CI [88.64% to 95.98%], *I*^2^ = 94.37%, *P* = .462) (Fig. 3).

### 3.5. Opioid requirements 48 hours post-operatively

**Figure 4.**
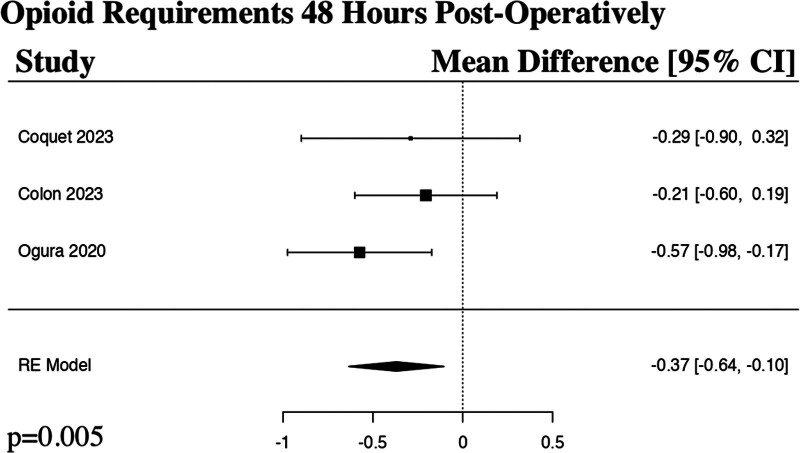
Forest plot demonstrating random effects model for opioid requirements 48 hours post-operatively in patients who received a regional anesthetic block in comparison to controls for anterior lumbar interbody fusion spine surgery. CI = confidence interval, RE = random effects.

The meta-analysis for the opioid requirements at 48 hours post-operatively included 3 studies.^[18–20]^ The results of the pooled analysis showed that a preoperative regional anesthetic block resulted in a statistically significant decrease in opioid requirements at 48 hours post-operatively in comparison to controls in patients undergoing ALIF surgery (MD −0.37, 95% CI [−0.63 to −0.11], PI [−0.63 to −0.12], *I*^2^ = 6.38%, *I*^2^ 95% CI [0.00% to 63.01%], *P *= .005) (Fig. 4).

### 3.6. Opioid requirements 72 hours post-operatively

**Figure 5.**
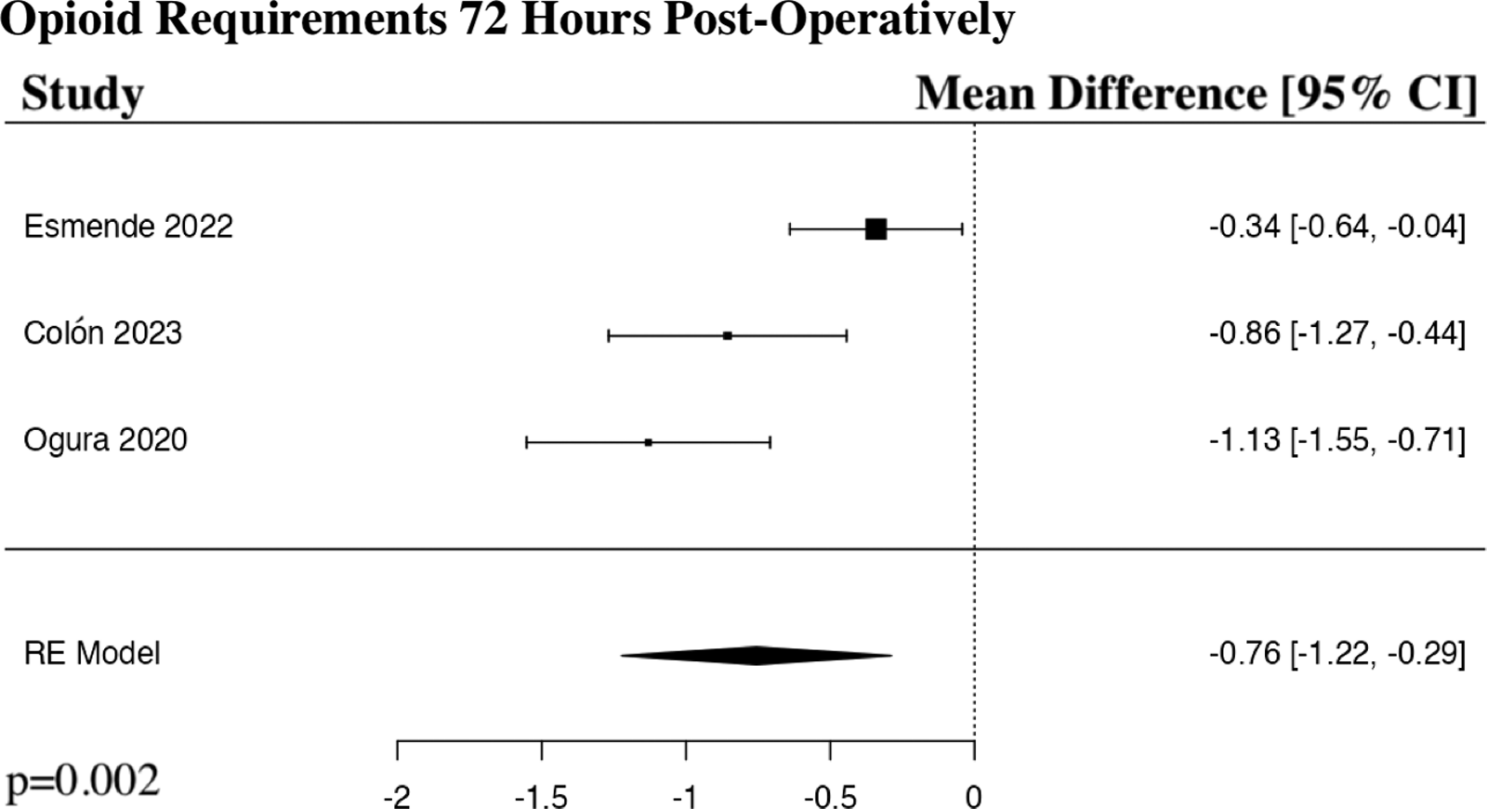
Forest plot demonstrating random-effects model for opioid requirements 72 h post-operatively in patients who received a regional anesthetic block in comparison to controls for anterior lumbar interbody fusion spine surgery. CI = confidence interval, RE = random effects.

The meta-analysis for the opioid requirements at 72 hours post-operatively included 3 studies.^[17,18,20]^ The results of the pooled analysis showed that a preoperative regional anesthetic block resulted in a statistically significant decrease in opioid requirements at 72 hours post-operatively in comparison to controls in patients undergoing ALIF surgery (MD −0.76, 95% CI [−1.22 to −0.29], PI [−1.66 to 0.14], *I*^2^ = 78.48%, *I*^2^ 95% CI [0.00% to 89.49%], *P* = .002) (Fig. 5).

## 4. Discussion

Our study is the first systematic review with meta-analyses to study the safety and efficacy of regional anesthetic blocks in patients undergoing ALIF surgery.

The results of our systematic review suggest that anterior regional anesthetic blocks via TAP or rectus sheath blocks are reasonably safe. Despite the included studies not comparing the total number of adverse events in each study group, no specific adverse events were reported due to the regional anesthetic block, such as blood reflux, digestive fluid aspirate, or local anesthetic intoxication. Coquet et al compared postoperative nausea/vomiting between groups and found a similar incidence between groups with 3/21 patients in the block group experiencing nausea/vomiting in comparison to 4/21 patients in the control group.^[19]^ Ogura et al compared postoperative ileus between the block and control groups and found a decreased incidence of postoperative ileus in the block group 5.88% (4/68) compared to the control group 15.38% (6/39), though not significantly different (*P* = .102).^[20]^

The results of our meta-analyses show a statistically significant decrease in postoperative opioid requirements at 48 hours (*P* = .005) and 72 hours post-operatively (*P* = .002) in the regional anesthetic block groups in comparison to controls. Interestingly, our study demonstrated a decrease in postoperative opioid requirements (*P* = .462) and numerical pain rating scores at 24 hours (*P* = .116) in the regional anesthetic block group in comparison to controls, though not to a level of statistical significance. Rather than the typical expected effect of a regional anesthetic block rapidly losing effect over time, these results instead suggest a somewhat delayed response in maximal benefit. The reliability of comparison between groups would be strengthened in future studies if the activity at the time of numerical pain score is also standardized, for example, documenting if the patient is at rest versus ambulating. Esmende et al was the only study that sub-analyzed the numerical pain rating scores when ambulating and at rest 24 hours post-operatively and found a statistically significant decrease in pain both with activity (*P* = .001) and at rest (*P* = .032) in the block group in comparison to the control group.^[17]^

Overall, the results of this study suggest that regional anesthetic blocks may be safe and effective analgesic adjuncts in the setting of ALIF surgery. However, these results should prompt further study into the analgesic efficacy of anterior abdominal regional anesthetic blocks. Special attention in future studies should be given to parsing out the ideal technique, medication, and dose. Additionally, parameters such as operative time, hospital length of stay, and patient satisfaction should be included in future studies. While there is an expected amount of time that must be dedicated to an experienced anesthesiologist performing the regional block, when studied by Esmende^[17]^ and Ogura et al,^[20]^ there was actually a decrease in operative times in each study, (*P* = .055) and (*P* = .071) respectively, though not to a level of statistical significance (Table 5). Coquet^[19]^ and Colon et al^[18]^ investigated hospital length of stay which was the same between groups at 4 days in each study arm. There are currently 2 ongoing clinical trials investigating preoperative TAP blocks for ALIF surgery whose results should help to further inform future providers of its safety and efficacy.^[21,22]^

**Table 5:** Notable results of studies included in meta-analyses.

Study	N Block	N Control	Results
Esmende^[17]^	88	87	• Block group had a decrease in PACU care time (*P* = .007) • Block group had a decrease in 72 h opioid consumption (*P* = .026) • Block group had a decrease in pain at rest (*P* = .032) and with activity (*P* = .001) • No significant difference in OR time, hospital LOS, 30-d ED visits, 30-day readmissions or unplanned return to OR
Colon^[18]^	47	52	• Block group had a decrease in opioid consumption on POD 0 (*P* = .004) • Block group had a decrease in cumulative opioid consumption on POD 0–2 (*P* = .036), POD 0–5 (*P* = .05) • Block group had a decrease in VAS scores on POD 3 (*P* = .011), POD 4 (*P* = .008) • No significant difference in hospital LOS or complication rates
Coquet^[19]^	18	16	• No significant difference in opioid consumption at 24 h post-operatively (*P* = .503) • No significant difference in opioid related side effects or hospital LOS
Ogura^[20]^	68	39	• Block group had a decrease in cumulative opioid consumption on POD 1 (*P* = .006), POD 2–7 (*P* < .001) • No significant difference in postoperative ileus, hospital LOS, hospital cost

ED = emergency department, LOS = length of stay, N = number, OR = operating room, PACU = post-anesthesia care unit, POD = postoperative day, VAS = visual analog scale.

This study is limited by the pooled data from the relatively small series included in the analyses, many of which were retrospective in design. The quality of the included retrospective studies was quite good but with some limitations, including a lack of standardized anterior regional block technique, type of medication administered in the block, or dose. Furthermore, the control interventions, baseline postoperative analgesia employed, and follow-up were heterogeneous among studies. Additionally, there was heterogeneity concerning the technical details of the surgeries among studies, specifically, the number of spinal levels fused and whether the construct was supplemented with posterior instrumentation. Given the limited literature on this topic, it is feasible that it may suffer from publication bias.

The results of this systematic review with meta-analyses suggest that in patients undergoing ALIF surgery, a preoperative regional anesthetic block is reasonably safe and results in a statistically significant decrease in opioid requirements at 48 and 72 hours post-operatively in comparison to controls. Additionally, a preoperative regional anesthetic block resulted in a reduction in opioid requirements as well as numerical pain rating scores at 24 hours post-operatively in comparison to controls, though not to a level of statistical significance. More robust prospective randomized studies are necessary to help inform the safety and efficacy of preoperative regional anesthetic blocks for ALIF surgery.

## Acknowledgments

Roman Haynatzki, Department of Bioinformatics at the University of Nebraska Medical Center, for reviewing statistical methods.

## Author contributions

**Conceptualization:** Luke J. Weisbrod, Anthony J. Maxin, Andrew P. Gard, Daniel L. Surdell.

**Data curation:** Luke J. Weisbrod, Anthony J. Maxin, Judith R. Bergjord.

**Formal analysis:** Luke J. Weisbrod, Anthony J. Maxin, Daniel L. Surdell.

**Investigation:** Luke J. Weisbrod, Anthony J. Maxin, Daniel L. Surdell.

**Methodology:** Luke J. Weisbrod, Anthony J. Maxin, Andrew P. Gard, Daniel L. Surdell.

**Project administration:** Luke J. Weisbrod, Anthony J. Maxin.

**Resources:** Luke J. Weisbrod, Anthony J. Maxin, Daniel L. Surdell.

**Software:** Luke J. Weisbrod, Anthony J. Maxin.

**Supervision:** Luke J. Weisbrod, Judith R. Bergjord, Andrew P. Gard, Daniel L. Surdell.

**Validation:** Luke J. Weisbrod, Anthony J. Maxin, Daniel L. Surdell.

**Visualization:** Luke J. Weisbrod, Anthony J. Maxin, Daniel L. Surdell.

**Writing – original draft:** Luke J. Weisbrod, Anthony J. Maxin, Andrew P. Gard, Daniel L. Surdell.

**Writing – review & editing:** Luke J. Weisbrod, Anthony J. Maxin, Judith R. Bergjord, Andrew P. Gard, Daniel L. Surdell.
